# Microbiome Disturbance, Nutritional Vulnerability, and Treatment Tolerance in Pancreatic Ductal Adenocarcinoma: Mechanistic Links and Clinical Readiness

**DOI:** 10.3390/cancers18162658

**Published:** 2026-08-17

**Authors:** Naotake Funamizu, Yasutaka Ihara, Kei Tamura, Yoshiaki Kamei, Yuzo Umeda

**Affiliations:** 1 Department of Hepatobiliary Pancreatic and Breast Surgery, Ehime University Graduate School of Medicine, Toon 791-0295, Ehime, Japan; kei58221@gmail.com (K.T.); kamei.yoshiaki.mz@ehime-u.ac.jp (Y.K.); umeda.yuzo.oe@ehime-u.ac.jp (Y.U.); 2 Department of Gastroenterological Surgery for Advanced Regional Healthcare, Ehime University Graduate School of Medicine, Toon 791-0295, Ehime, Japan; 3 Clinical Research Promotion Unit, Clinical Therapeutic Trial Center, Ehime University Hospital, Toon 791-0295, Ehime, Japan; ihara.yasutaka.gr@ehime-u.ac.jp

**Keywords:** pancreatic ductal adenocarcinoma, gut microbiota, microbiome, dysbiosis, nutritional vulnerability, pancreatic exocrine insufficiency, cachexia, sarcopenia, frailty, treatment tolerance, clinical readiness

## Abstract

**Background/Objectives:** Pancreatic ductal adenocarcinoma (PDAC) is characterized by aggressive tumor biology and profound host vulnerability, including pancreatic exocrine insufficiency (PEI), maldigestion, malnutrition, cachexia, sarcopenia, frailty, systemic inflammation, and poor tolerance to multimodal therapy. Gut and intratumoral microbiota have been implicated in pancreatic carcinogenesis, tumor immunity, chemotherapy response, and postoperative outcomes. However, the clinical readiness of microbiome-informed supportive care in PDAC remains uncertain. **Results:** Current evidence supports plausible mechanistic links among PEI, maldigestion, dysbiosis, microbial metabolites, barrier dysfunction, systemic inflammation, cachexia, sarcopenia, and treatment intolerance. Nevertheless, PDAC microbiome research is limited by major heterogeneity in sampling sites, sequencing platforms, antibiotic exposure, biliary drainage, diet, treatment timing, tumor stage, and analytic pipelines. Evidence is also discordant, particularly regarding alpha diversity and reproducible microbial signatures. Low-biomass tissue contamination and incomplete consideration of fungal and multi-kingdom microbiota further limit interpretation. **Conclusions:** Microbiome disturbance should currently be viewed as an investigational modifier of nutritional vulnerability and treatment tolerance rather than as a validated clinical biomarker or therapeutic target in PDAC. A clinically responsible framework should distinguish what is actionable now—nutrition screening, PEI management, inflammation and frailty assessment, body-composition evaluation, and treatment-exposure monitoring—from what remains investigational, including microbiome profiling, microbial signatures, probiotics, prebiotics, fecal microbiota transplantation, and metabolite-guided intervention.

## Simple Summary

Pancreatic ductal adenocarcinoma is often accompanied by pancreatic exocrine insufficiency, maldigestion, weight loss, cachexia, sarcopenia, frailty, and systemic inflammation. These host factors reduce the ability of patients to tolerate surgery and anticancer therapy. Gut and tumor-associated microorganisms may also influence pancreatic cancer biology, but current microbiome findings are heterogeneous and are not yet ready for routine treatment selection. This critical review reframes the pancreatic cancer microbiome literature from an oncology supportive-care perspective. Rather than proposing immediate microbiome-directed therapy, we examine how microbial disturbance may interact with nutritional vulnerability, pancreatic exocrine insufficiency, inflammation, cachexia, sarcopenia, and treatment tolerance. The current clinical priority is careful assessment and management of nutrition, frailty, body composition, pancreatic exocrine insufficiency, and treatment-related stress, together with careful documentation and judicious management of biliary drainage and antibiotic exposure, while microbiome profiling remains primarily a research tool.

## 1. Introduction

Pancreatic ductal adenocarcinoma (PDAC) remains one of the most lethal solid malignancies, with both incidence and mortality continuing to rise [1]. In addition to aggressive tumor biology, PDAC is defined by profound host vulnerability. Pancreatic exocrine insufficiency (PEI), maldigestion, weight loss, malnutrition, cachexia, sarcopenia, frailty, systemic inflammation, and impaired functional reserve frequently coexist and may compromise perioperative recovery, adjuvant chemotherapy completion, systemic therapy tolerance, quality of life, and survival [2,3,4]. These problems are also supported by studies and guidelines addressing frailty, nutritional risk, and cancer cachexia [5,6,7]. Additional supportive-care guidance emphasizes the clinical importance of nutrition and cachexia management [8,9,10]. Contemporary treatment of PDAC is based primarily on multimodal cytotoxic chemotherapy, including FOLFIRINOX-based regimens, gemcitabine-based combinations, and NALIRIFOX in advanced disease [11]. At the same time, molecularly selected therapies are becoming increasingly relevant for small subsets of patients with actionable alterations. These include strategies targeting DNA damage repair deficiencies, particularly BRCA1/2- or PALB2-associated tumors; maintenance olaparib represents an established precision-treatment approach for patients with germline BRCA-mutated metastatic PDAC whose disease has not progressed during platinum-based chemotherapy [12]. Rapidly evolving approaches directed at KRAS and downstream signaling pathways are also being investigated, with recent early-phase studies demonstrating emerging clinical activity of KRAS-targeted agents in pancreatic cancer [13]. Immune-modulatory and tumor-microenvironment-directed strategies remain under active investigation, although their clinical benefit remains limited in unselected PDAC. As treatment options become increasingly intensive and biologically individualized, maintaining nutritional and functional reserve and treatment tolerance becomes increasingly important. In parallel, accumulating evidence suggests that gut and tumor-associated microbiota may contribute to pancreatic carcinogenesis, tumor immune microenvironment, chemotherapy response, and postoperative outcomes. Fecal and metagenomic studies have reported PDAC-associated microbial signatures [14,15,16]. Systematic and translational studies further support a role for gut or tissue-associated microbial communities in PDAC biology [17,18,19]. Experimental and clinical outcome studies have also linked tumor-associated microbiota to immune regulation and prognosis [20,21,22]. However, microbiome research in PDAC has often been presented as a diagnostic or therapeutic frontier without adequate attention to clinical confounding, host nutritional status, PEI, biliary drainage, antibiotic exposure, and treatment timing. These factors are not peripheral; they are central to the interpretation of microbiome data in pancreatic cancer.

Several reviews have addressed pancreatic cancer and the gut microbiome, including a recent systematic review that framed microbiota as a precision-medicine approach for pancreatic cancer management [23]. Other reviews have addressed dietary recommendations, cachexia-related microbiome changes, and nutrition/gut health in PDAC [24,25,26]. Additional reviews have focused on PEI, microbiome-based biomarkers, and microbiota as a therapeutic target [27,28,29]. Broader reviews have also summarized pancreatic microbiota in health and disease [30]. The present review differs by focusing on clinical readiness and by positioning microbiome disturbance as an investigational modifier of nutritional vulnerability and treatment tolerance rather than as an immediately actionable biomarker. Its added value is therefore not another microbiome discovery summary, but a critical oncology supportive-care synthesis centered on clinical readiness, confounding, host vulnerability, and treatment tolerance.

This framing is important because the strongest current clinical implication of the microbiome literature is not routine microbiome testing or microbiome manipulation. Rather, microbiome-informed thinking should reinforce systematic assessment of modifiable host factors, including PEI, maldigestion, malnutrition, systemic inflammation, sarcopenia, frailty, body composition, biliary drainage, antibiotic exposure, and treatment-related gastrointestinal stress. Against this background, this review examines mechanistic links, methodological caveats, contradictory evidence, mycobiome considerations, and clinical readiness in the microbiome–nutrition–treatment tolerance axis in PDAC. This narrative review prioritizes human PDAC evidence where available and uses preclinical and non-PDAC studies primarily to provide mechanistic context. The distinctive position of the present review relative to closely related reviews is summarized in Table 1.

**Table 1:** Conceptual position of the present review relative to closely related reviews.

Review or Topic Area	Primary Focus	Limitation from the Perspective of Clinical Readiness	Distinctive Contribution of the Present Review
PDAC gut microbiome reviews [23,24,28,29,30]	Microbial signatures, carcinogenesis, immune modulation, biomarkers, microbiota-based precision medicine, and microbiome-targeted therapy; recent precision-medicine-oriented systematic reviews	Often emphasize microbiome discovery more than host vulnerability, PEI, sarcopenia, frailty, or treatment tolerance	Reframes microbiome disturbance as an investigational modifier of nutritional vulnerability and treatment tolerance, with emphasis on clinical readiness rather than microbiome-directed treatment selection
Nutrition and gut health reviews in PDAC [26]	Diet, malnutrition, nutritional assessment, and translational nutrition	Microbiome is usually discussed contextually rather than as part of treatment tolerance and clinical readiness	Links nutritional vulnerability to microbial metabolites, inflammation, PEI, cachexia, sarcopenia, and therapy tolerance
Cachexia-microbiome reviews [25]	Cancer cachexia, inflammation, microbial metabolites, and experimental intervention	Often not PDAC-specific and may not integrate biliary drainage, PEI, surgery, and adjuvant therapy completion	Applies cachexia-microbiome concepts to PDAC-specific digestive and perioperative vulnerability
PEI-microbiome reviews [27]	PEI, maldigestion, enzyme replacement, and gut microbial alteration	PEI may be treated as a separate digestive problem rather than part of broader oncology treatment resilience	Extends PEI to a wider clinical framework involving nutrition, frailty, inflammation, body composition, and treatment exposure
Present review	Microbiome disturbance, nutritional vulnerability, treatment tolerance, and clinical readiness in PDAC	Not applicable	Distinguishes actionable supportive care from investigational microbiome-based strategies

## 2. Nutritional Vulnerability and Treatment Tolerance in PDAC

Nutritional vulnerability is not a secondary problem in PDAC; it is a central determinant of oncology treatment tolerance. PEI, maldigestion, reduced dietary intake, steatorrhea, micronutrient deficiency, systemic inflammation, and catabolic tumor biology may converge to accelerate weight loss and cachexia [2,3,8]. Cachexia and nutrition guidelines further support early identification and management of these problems [9,10]. In advanced or unresectable pancreatic cancer, PEI and malnutrition have also been linked to chemotherapy-course vulnerability and impaired supportive-care outcomes [31,32,33]. Sarcopenia, myosteatosis, and frailty provide complementary information beyond body mass index and body weight, and they may help identify patients with reduced physiological reserve [4,5]. Additional studies have linked frailty, sarcopenia, body composition, and nutritional indices to outcomes after pancreatic surgery or in pancreatic cancer [7,34,35]. Similar associations have been reported in studies of skeletal muscle mass, frailty, and GNRI [36,37,38]. An HBP-specific frailty framework also supports this broader interpretation [39].

From a surgical oncology perspective, these host factors are clinically important because they may influence postoperative morbidity, infection risk, delayed recovery, reduced relative dose intensity, failure to complete adjuvant chemotherapy, and poor long-term outcomes. Nutritional risk and chemotherapy-completion studies support this treatment-tolerance framework [6,7,40]. Frailty and nutritional-risk studies after pancreatic surgery provide complementary evidence [37,38,39]. In resectable PDAC, nutritional indices, systemic inflammation markers, and frailty indices are clinically practical because they can be measured before treatment and can be repeated during perioperative and adjuvant therapy periods [40].

This treatment-tolerance framework is the appropriate clinical anchor for interpreting microbiome disturbance in PDAC. Microbiota-related findings should not be considered in isolation from PEI, biliary obstruction, biliary drainage, antibiotic exposure, diabetes mellitus, cachexia, and treatment phase. Instead, the microbiome should be viewed as one layer within a complex host–tumor–digestive ecosystem that may modify nutritional vulnerability and treatment resilience.

## 3. Microbiome Disturbance in PDAC: Reproducible Patterns and Unresolved Questions

In some studies, human PDAC has been associated with gut microbial dysbiosis, including reduced diversity, enrichment of oral or inflammation-associated taxa, depletion of short-chain fatty acid (SCFA)-producing bacteria, and changes in microbial metabolic pathways [14,15,16]. However, these findings are not uniformly reproduced across cohorts, and systematic synthesis has emphasized inconsistency in some diversity metrics [17]. In parallel, tumor-associated microbiota have been reported in PDAC, with experimental and translational studies suggesting potential links to immune suppression, chemotherapy metabolism, and patient outcomes [19,20,21]. Additional experimental work supports microbiota-related immune modulation in pancreatic tumor models [22].

However, reproducibility at the level of individual taxa remains limited. Reported increases in taxa such as Streptococcus, Veillonella, Klebsiella, Enterobacteriaceae, Fusobacterium, or Porphyromonas and decreases in Faecalibacterium, Roseburia, Bifidobacterium, or other SCFA-producing bacteria vary by cohort, geography, analytic pipeline, disease stage, treatment exposure, and sample type. This heterogeneity is evident across fecal, metagenomic, and systematic-review studies [14,15,16]. It is also apparent in more recent systematic and tissue-focused analyses [17,18]. Therefore, current evidence is more reliable for broad ecological or functional disturbance than for a single validated microbial signature. Representative original studies relevant to microbiome disturbance, PDAC biology, treatment response, and clinical outcomes are summarized in Table 2.

**Table 2:** Representative original studies relevant to microbiome disturbance, PDAC biology, treatment response, and clinical outcomes.

Study	Sample/Method	Cohort Size/Setting	Main Findings	Key Limitations
Kartal et al., 2022 [15]	Feces, saliva, selected pancreatic tissue; shotgun metagenomics and 16S rRNA sequencing	57 PDAC, 50 controls, 29 chronic pancreatitis; external German validation cohort	A 27-species fecal signature identified PDAC; performance improved with CA19-9 and was externally validated.	Primarily diagnostic; geographic, dietary, and clinical-exposure effects may limit reproducibility.
Geller et al., 2017 [19]	PDAC tumor tissue and bacterial isolates; microbial characterization and functional drug-metabolism assays	113 PDAC tumors; mechanistic gemcitabine-resistance study	Intratumoral bacteria could metabolize gemcitabine and induce resistance in experimental systems.	Partly preclinical; low-biomass contamination concern; clinical benefit remains unproven.
Riquelme et al., 2019 [21]	PDAC tumor tissue; stool for experimental FMT; 16S rRNA sequencing	68 resected PDAC tumors; long- vs. short-term survivors	Higher tumor microbial diversity and a specific signature were associated with long-term survival and immune infiltration.	Retrospective design; modest cohort; low-biomass contamination and causal inference remain concerns.
Tintelnot et al., 2023 [41]	Feces and serum; shotgun metagenomics, metabolomics, functional FMT	Metastatic PDAC receiving chemotherapy; small human discovery and validation cohorts	Microbiota-derived 3-IAA was associated with chemotherapy response and enhanced treatment efficacy experimentally.	Small human cohorts; substantial mechanistic evidence from animal models; prospective validation required.
Stein-Thoeringer et al., 2025 [42]	Bile, upper-GI, and operative-site samples; 16S rRNA sequencing and microbial culture	101 patients undergoing pancreatic head resection; 50 PDAC	Enterococcus-associated dysbiosis was linked to severe surgical-site infection and higher mortality.	Single-center cohort; multiple clinical exposures may confound associations.

For clinical interpretation, this distinction is crucial. Microbial disturbance may be biologically relevant to PDAC, but it is not yet a diagnostic, prognostic, or therapeutic biomarker ready for routine oncology practice. Its main value at present is to generate hypotheses about mechanisms that connect maldigestion, inflammation, immune modulation, cachexia, sarcopenia, and treatment intolerance.

## 4. Methodological Heterogeneity and Contamination: Why Cross-Study Comparability Is Difficult

Methodological heterogeneity is one of the most important barriers to clinical translation of PDAC microbiome research. Studies differ in sampling site, including stool, saliva, duodenal fluid, bile, pancreatic juice, tumor tissue, adjacent normal tissue, and postoperative drainage fluid. These biospecimens reflect different ecological compartments and cannot be assumed to represent the same microbial community.

Sequencing platforms further complicate interpretation. 16S rRNA gene sequencing, shotgun metagenomic sequencing, metatranscriptomics, metabolomics, quantitative PCR, and culture-based approaches differ in taxonomic resolution, functional inference, ability to detect strain-level variation, and sensitivity to contamination. Analytic pipelines, reference databases, DNA extraction protocols, batch effects, and statistical correction methods may substantially influence results.

Clinical confounding is particularly severe in PDAC. Biliary obstruction, endoscopic biliary drainage, cholangitis, perioperative antibiotics, diabetes mellitus, PEI, pancreatic enzyme replacement therapy, diet, proton-pump inhibitor exposure, neoadjuvant therapy, chemotherapy, surgery, fasting, postoperative anatomy, and tumor stage may all alter microbial communities. Recent clinical studies also suggest that antibiotic exposure and upper-gastrointestinal Enterococcus-associated dysbiosis may be related to outcomes in surgically treated PDAC [42,43]. Without careful measurement and adjustment for these factors, microbiome signatures may reflect clinical exposures rather than cancer-specific biology.

Low-biomass contamination is especially important for pancreatic tissue and tumor microbiome studies. Because pancreatic tissue contains relatively low microbial biomass, signals from reagents, instruments, laboratory environment, surgical contamination, or sequencing background may be misinterpreted as tissue-resident microbiota. This problem has been emphasized in foundational microbiome-methodology studies showing that reagent and laboratory contamination can critically affect sequence-based analyses, particularly when microbial biomass is low [44,45]. Rigorous negative controls, decontamination analyses, microbial load quantification, spatial validation, and independent replication are therefore required before intratumoral microbiota findings can be interpreted as biologically meaningful. The major sources of methodological and clinical heterogeneity in PDAC microbiome studies are summarized in Table 3.

**Table 3:** Major sources of heterogeneity in PDAC microbiome studies and their implications for clinical readiness.

Domain	Examples	Interpretative Problem	Recommended Reporting or Control
Sampling site	Stool, bile, pancreatic juice, tumor tissue, duodenal fluid, saliva	Different compartments reflect different microbial ecosystems	Report biospecimen type, collection timing, storage, and anatomical context
Sequencing method	16S rRNA, shotgun metagenomics, metatranscriptomics, qPCR, culture	Different taxonomic and functional resolution	Specify platform, region, database, pipeline, and quality-control methods
Analytic pipeline and batch effects	DNA extraction method; sequencing depth; reference database; taxonomic classifier; batch effects; statistical correction	Apparent taxa-level signatures may reflect technical processing rather than biological differences	Report pipeline details, include batch controls, and apply appropriate normalization and sensitivity analyses
Clinical exposure	Antibiotics, biliary drainage, cholangitis, PPI, chemotherapy, surgery	Microbial shifts may reflect treatment or procedures rather than PDAC biology	Record and adjust for exposure timing and duration
Nutritional state	PEI, malnutrition, cachexia, sarcopenia, diet, PERT	Nutrition may be both cause and consequence of dysbiosis	Measure PEI, diet, body composition, frailty, inflammation, and enzyme therapy
Low-biomass contamination	Tumor tissue, normal pancreas, intrapancreatic specimens	False-positive microbial signals may occur	Use negative controls, microbial load estimation, decontamination, and spatial validation

## 5. Mechanistic Integration: PEI, Maldigestion, Dysbiosis, Inflammation, Cachexia, and Sarcopenia

**Figure 1:**
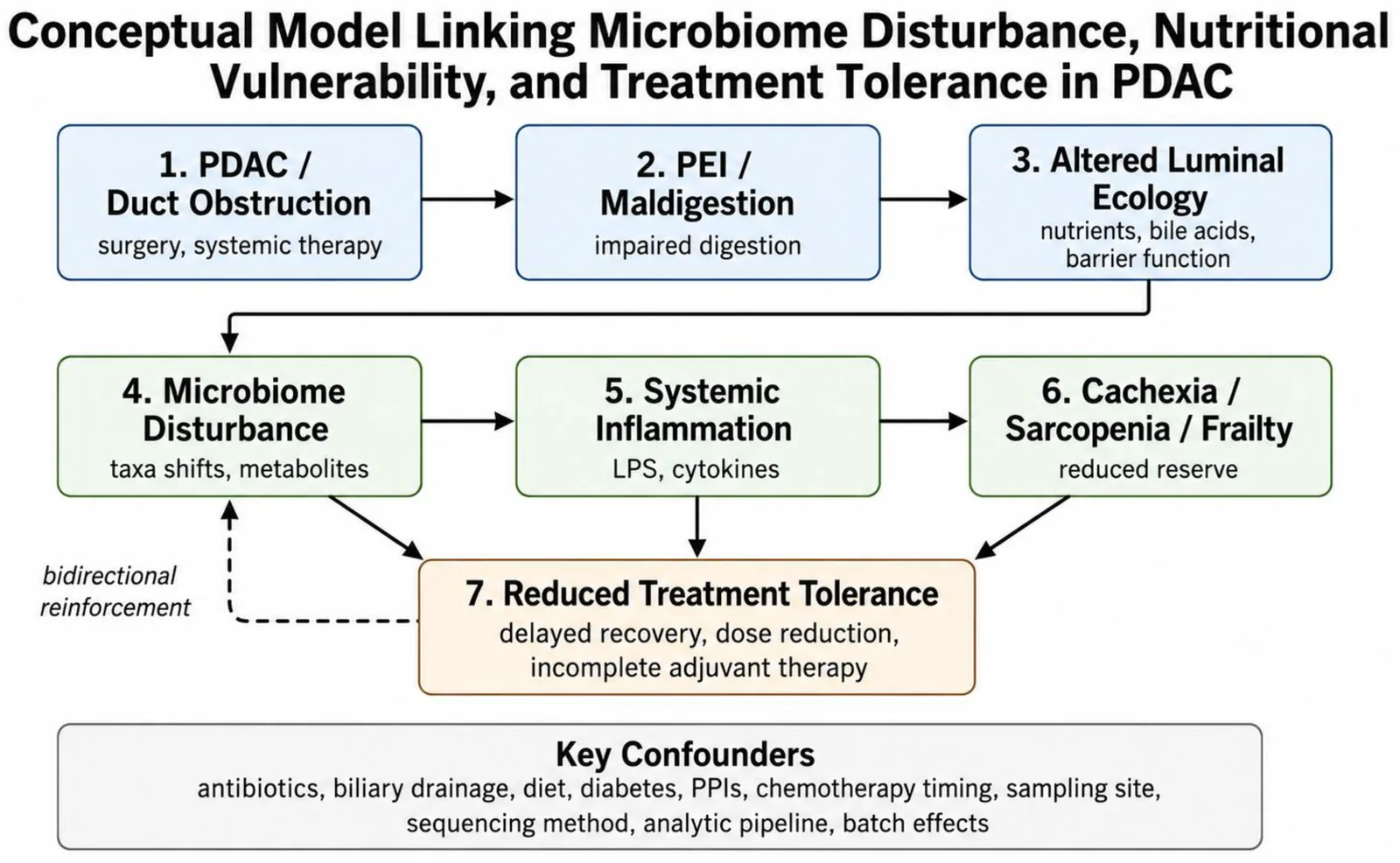
Conceptual model linking microbiome disturbance, nutritional vulnerability, and treatment tolerance in pancreatic ductal adenocarcinoma. Pancreatic duct obstruction, surgery, and systemic therapy may contribute to pancreatic exocrine insufficiency and maldigestion, leading to altered luminal ecology, including changes in nutrient availability, bile acids, and barrier function. These changes may interact with microbiome disturbance, systemic inflammation, cachexia, sarcopenia, and frailty, ultimately reducing treatment tolerance. Antibiotic exposure, biliary drainage, diet, diabetes, proton-pump inhibitor use, chemotherapy timing, sampling site, sequencing method, analytic pipeline, and batch effects are key confounders that should be documented in future studies. The arrows represent proposed or associative relationships based on clinical, translational, and preclinical evidence and should not be interpreted as established causal pathways in human PDAC. The model is intended as a clinical-readiness framework and does not imply routine microbiome-guided treatment selection. Blue boxes represent disease- and digestion-related factors, green boxes represent microbiome- and host-related biological processes, the orange box represents reduced treatment tolerance, and the gray box represents major confounding factors. Solid arrows indicate the proposed directional relationships among the components, whereas the dashed arrow indicates potential bidirectional reinforcement between microbiome disturbance and reduced treatment tolerance.

A clinically meaningful integration of microbiota and nutritional vulnerability requires a bidirectional mechanism rather than a parallel description. PEI and maldigestion may alter luminal substrate availability, fat digestion, bile acid handling, intestinal transit, and microbial metabolism [2,3]. In turn, dysbiosis may influence mucosal barrier function, microbial metabolite production, systemic inflammation, immune regulation, and host metabolism [41,46,47]. This integrated pathway is summarized in Figure 1.

One plausible pathway begins with PEI and maldigestion, leading to altered luminal nutrient availability and bile acid exposure, which may promote microbial disturbance. This disturbance may reduce SCFA production and alter bile acid signaling, thereby contributing to mucosal barrier dysfunction and systemic inflammation and, ultimately, to cachexia, sarcopenia, and reduced treatment tolerance. An alternative pathway begins with dysbiosis, which may increase lipopolysaccharide exposure and TLR-mediated inflammatory signaling, promoting systemic inflammation and catabolism with subsequent nutritional deterioration and loss of functional reserve [46,47,48]. A murine cachexia model supports links among cachexia, gut microbiota composition, and SCFA metabolism [49], while experimental pancreatic cancer data demonstrate that lipopolysaccharide-related signaling can enhance invasive tumor behavior [50]. Although these pathways have not been fully validated in human PDAC cohorts, they provide a biologically plausible mechanistic bridge between microbiome research and supportive oncology.

Tumor-associated bacteria may further influence treatment response through immune modulation or drug metabolism. Experimental studies have shown that intratumoral bacteria can metabolize gemcitabine under specific conditions [19,51], while tumor microbial diversity has been associated with immune infiltration and long-term survival in selected PDAC cohorts [21]. Additional experimental and translational studies using pancreatic tumor-associated bacteria support the biological plausibility of functional tumor microbiota [52,53]. However, these findings remain largely associative or mechanistic, and their clinical interpretation is limited by the potential for low-biomass contamination and by the absence of prospective evidence showing that microbiome-directed intervention improves treatment outcomes.

Microbial metabolites may represent a more clinically tractable interface between microbial disturbance and host vulnerability than individual taxa-level signatures. SCFAs, secondary bile acids, and tryptophan-derived metabolites can influence epithelial barrier integrity, immune regulation, systemic metabolism, and chemotherapy response [41,46,47]. In PDAC, these metabolite pathways may eventually complement established assessments of nutritional status, PEI, systemic inflammation, and functional reserve. However, prospective clinical validation is required before microbiome- or metabolite-based markers can be used to guide patient-specific treatment or supportive-care interventions.

## 6. Treatment Tolerance and Oncology Supportive Care

The clinical question is not simply whether PDAC is associated with dysbiosis. The more relevant question is whether microbiome-related information improves prediction, prevention, or management of treatment intolerance beyond established clinical assessments. At present, there is insufficient evidence to recommend routine stool profiling, tumor microbiome testing, probiotics, prebiotics, antibiotics, fecal microbiota transplantation, or metabolite-guided therapy for unselected patients with PDAC.

However, the microbiome literature can refine supportive-care priorities. Patients with PEI, biliary drainage, repeated antibiotic exposure, malnutrition, sarcopenia, frailty, high systemic inflammation, or poor oral intake may represent high-risk phenotypes in whom microbial disturbance and nutritional vulnerability are likely to cluster. In these patients, the actionable steps remain standard but should be performed systematically: nutritional screening, pancreatic enzyme replacement when indicated, symptom management, inflammation assessment, body-composition evaluation, frailty assessment, physical and nutritional prehabilitation when feasible, and longitudinal monitoring during chemotherapy.

Thus, the clinical framework should be presented as “clinical readiness” rather than “microbiome-driven precision care”. Microbiome testing may be incorporated into prospective research protocols, but routine clinical management should not depend on unvalidated microbial cutoffs or taxa-level signatures.

## 7. Contradictory Evidence and Limits of the Dysbiosis Paradigm

A balanced review must address contradictory evidence. Although several studies report reduced microbial diversity in PDAC, systematic syntheses have shown that alpha diversity differences between PDAC and healthy controls are not always consistent [17]. Beta diversity, taxa-level enrichment, and machine-learning signatures may also vary across cohorts and may be affected by geography, diet, sequencing method, diabetes, biliary drainage, and antibiotic exposure.

The term “dysbiosis” is therefore useful but imprecise. It can imply a disease-specific microbial abnormality even when observed differences may reflect malnutrition, cholestasis, PEI, treatment exposure, or methodological bias. In PDAC, dysbiosis should be interpreted as an ecological and functional disturbance occurring within a complex host–tumor–digestive context, not as a single reproducible microbial pattern.

These limitations do not invalidate the microbiome field. Rather, they define the next step. Future studies should move beyond case–control description and should include longitudinal sampling, standardized biospecimen handling, exposure recording, nutritional and frailty assessment, PEI evaluation, metabolomics, inflammatory markers, and externally validated clinical outcomes such as postoperative complications, dose intensity, adjuvant chemotherapy completion, and survival.

## 8. Mycobiome and Multi-Kingdom Microbiota

Most PDAC microbiome studies have focused on bacteria. However, the fungal microbiome, or mycobiome, may also be relevant to pancreatic oncogenesis and immune regulation. Experimental evidence has suggested that fungal communities may promote pancreatic oncogenesis through complement-related immune pathways, including mannose-binding lectin activation [54]. Immune consequences of fungal dysbiosis have also been described in non-PDAC mucosal models, supporting multi-kingdom caution without implying clinical actionability in PDAC [55].

The mycobiome remains far less developed clinically than the bacterial microbiome. Fungal biomass, contamination risk, extraction protocols, ITS sequencing methods, and limited reference databases complicate interpretation. Nevertheless, omission of the mycobiome may lead to an incomplete view of the tumor and gut microbial ecosystem. Future PDAC studies should consider multi-kingdom profiling, including bacteria, fungi, viruses, and metabolites, particularly when investigating immune regulation, cachexia, treatment tolerance, or microbiome-targeted intervention.

At present, mycobiome findings should be regarded as mechanistically informative but clinically investigational. They do not justify antifungal or microbiome-directed intervention outside research settings.

## 9. Clinical Readiness: What Is Actionable Now and What Remains Investigational

The most important practical distinction is between actionable supportive care and investigational microbiome translation. Actionable now are interventions supported by nutrition, cachexia, PEI, perioperative, and supportive oncology evidence: early nutrition screening, assessment of intake and weight loss, PEI evaluation, pancreatic enzyme replacement when indicated, management of steatorrhea and gastrointestinal symptoms, assessment of muscle mass and function, frailty screening, inflammation monitoring, prehabilitation when feasible, and careful documentation and judicious management of biliary drainage and antibiotic exposure [2,3,8]. Cancer cachexia and supportive oncology guidelines further support systematic nutritional and functional assessment [9,10].

Therefore, microbiome-informed supportive care should not be interpreted as microbiome-directed treatment selection at present, but as a framework for documenting host factors that may confound, mediate, or modify microbial signatures.

Investigational modifiers are microbiome-based risk stratification, stool or bile microbiome screening, tumor microbiome profiling, taxa-specific cutoffs, probiotics, prebiotics, synbiotics, antibiotics for microbiome modulation, fecal microbiota transplantation, and metabolite-guided intervention. These strategies may become clinically useful, but current data are insufficient to define which microbial signatures should trigger which intervention, which thresholds should be used, or whether microbiome-guided care improves treatment completion or survival.

Clinical readiness in PDAC microbiome research should therefore be graded. Mechanistic plausibility is moderate to strong for inflammation, immune regulation, microbial metabolites, and treatment response. Clinical reproducibility is limited. Actionability is currently low for microbiome testing itself but high for standard nutritional, PEI, frailty, and supportive care assessments. This distinction avoids overstating the field while still identifying a rational research agenda. For this narrative review, clinical readiness was qualitatively categorized by the maturity and reproducibility of clinical evidence, the degree of external validation, the feasibility and standardization of implementation, and the availability of evidence supporting routine clinical use. High readiness denotes approaches supported by established clinical evidence or guidelines and suitable for current clinical practice; moderate readiness indicates promising clinical evidence with remaining limitations in validation or standardization; and low readiness denotes predominantly exploratory, preclinical, heterogeneous, or insufficiently validated approaches. These categories are intended as a pragmatic interpretative framework rather than a formally validated scoring system. The clinical readiness of microbiome–nutrition concepts in PDAC is summarized in Table 4.

**Table 4:** Clinical readiness of microbiome–nutrition concepts in PDAC.

Concept	Evidence Status	Clinical Readiness	Current Recommendation
Nutrition screening, PEI evaluation, and PERT	Supported by clinical guidelines and supportive-care literature	High	Use routinely when clinically indicated
Sarcopenia, frailty, and body-composition assessment	Increasing clinical evidence for prognosis and treatment tolerance	Moderate to high	Incorporate into risk assessment and perioperative/adjuvant planning
Gut microbiome profiling	Associative and heterogeneous evidence	Low	Use mainly in research protocols
Tumor microbiome profiling	Mechanistically interesting but affected by low-biomass contamination	Low	Require rigorous controls and validation
Microbial metabolites	Biologically plausible; emerging translational evidence	Low to moderate	Develop as research biomarkers linked to nutrition, PEI, inflammation, and treatment outcomes
Probiotics/prebiotics/synbiotics/FMT	Limited PDAC-specific interventional evidence	Low	Do not use as routine PDAC-directed therapy outside trials
Mycobiome profiling	Preclinical and early translational evidence	Low	Consider in multi-kingdom research, not routine care

## 10. Future Research Agenda

Future research should prioritize longitudinal and clinically integrated designs. Ideal studies would collect stool, blood, dietary, PEI, body-composition, frailty, inflammatory, treatment-exposure, and clinical outcome data at standardized time points: before treatment, after biliary drainage, before surgery, after surgery, during adjuvant therapy, and at recurrence. Such designs would help distinguish baseline cancer-associated microbial patterns from treatment-induced microbial shifts.

Clinically meaningful outcomes should include postoperative complications, infectious complications, gastrointestinal toxicity, treatment delay, relative dose intensity, adjuvant chemotherapy completion, hospital readmission, quality of life, recurrence-free survival, and overall survival. Microbiome data should be integrated with metabolomics, bile acid profiling, inflammatory markers, PEI assessment, and nutritional indices rather than analyzed as an isolated omics layer.

Interventional studies should be staged carefully. Before microbiome-targeted interventions are tested broadly, observational studies should identify reproducible high-risk phenotypes and modifiable pathways. Trials of nutritional intervention, PEI management, prehabilitation, or synbiotics should include microbiome and metabolite endpoints as mechanistic readouts, while avoiding unsupported claims that microbiome modification itself is an established therapeutic strategy.

## 11. Conclusions

PDAC is characterized by profound nutritional and functional vulnerability, including PEI, maldigestion, malnutrition, cachexia, sarcopenia, frailty, systemic inflammation, and impaired treatment tolerance. Microbiome disturbance may plausibly interact with these host factors through barrier dysfunction, microbial metabolites, immune regulation, bile acid signaling, inflammation, and treatment-related stress.

However, the clinical readiness of microbiome-informed care in PDAC remains limited. Current evidence is constrained by methodological heterogeneity, low-biomass contamination, inconsistent alpha diversity findings, limited reproducibility of taxa-level signatures, and insufficient interventional data. The most responsible conclusion is that microbiome disturbance is an investigational modifier of nutritional vulnerability and treatment tolerance, not yet a validated clinical biomarker or therapeutic target. The actionable priority is systematic supportive care—nutrition, PEI, inflammation, sarcopenia, frailty, and treatment-exposure assessment—while prospective studies determine whether microbiome and metabolite profiling can improve risk stratification and outcomes.

## Data Availability

No new data were created or analyzed in this study. Data sharing is not applicable to this article.

## Abbreviations

PDAC, pancreatic ductal adenocarcinoma; PEI, pancreatic exocrine insufficiency; PERT, pancreatic enzyme replacement therapy; SCFA, short-chain fatty acid; LPS, lipopolysaccharide; TLR, Toll-like receptor; FMT, fecal microbiota transplantation; PPI, proton-pump inhibitor.
